# Supplementary material for: Effects of an Unripe Avocado Extract on Glycaemic Control in Individuals with Obesity: A Double-Blinded, Parallel, Randomised Clinical Trial
Source: Nutrients. 2023 Nov 17;15(22):4812. doi: 10.3390/nu15224812 (PMC10674186; doi:10.3390/nu15224812)
Supplement: Supplementary file 1 [file nutrients-15-04812-s001.zip › nutrients-2693865-supplementary.pdf]

Supplementary Table S1. Dietary assessment at 1-week before and 11-week after the avocado extract or placebo intervention in adults with obesity[1]

	Avocado extract group (n=25)		Placebo group (n=25)		
	**W-1 to 0**	**W 11 to 12**	**W-1 to 0**	**W11 to 12**	**p Value (Between group)**
**Diet intake**					
Energy intake (kj)	9025 ± 620	8382 ± 432	8556 ± 462	8832 ± 5.83	0.231
Carbohydrates (g)	227 ± 15	213 ± 14	218 ± 15.2	226± 18	0.203
Protein (g)	91.7 ± 6.0	87 ± 5.1	89.7 ± 5.27	95.1± 5.56	0.420
Total fat (g)	88.0 ± 8.0	81.7 ± 4.8	82.3 ± 5.25	82.9± 6.58	0.690
Saturated fat (g)	34.2 ± 3.5	29.5 ± 1.95	31.4 ± 2.12	30.6 ± 2.71	0.346
Trans fats (g)	1.49 ± 0.176	1.21± 1.08	1.29 ± 0.145	1.21 ± 0.139	0.510
Polyunsaturated fats (g)	12.1 ± 1.19	12.2 ± 1.35	11.1 ± 0.875	12.5 ± 1.34	0.851
Monounsaturated fats (g)	29.6 ± 3.22	27.9 ± 2.17	27.4 ± 2.21	28.1 ± 2.52	0.745
Fibre (g)	22.5 ± 1.56	21.7 ± 1.32	21.5 ± 1.58	24.3 ± 2.97	0.454
**Sleep and physical activity**					
Total sleep time (hours)	7.96 ± 0.23	7.91 ± 0.18	8.39 ± 0.22	8.43 ± 0.22	0.712
Steps/day	7920 ± 659	7842 ± 789	7221 ± 922	7262 ± 729	0.944

[1] Data are presented as mean ± SEM. AvX, Avocado extract group. Between-group analysis for the change from Week 0 to Week 12 for each variable was performed using one-way ANCOVA, adjusted for baseline value and sex.

Figure S1

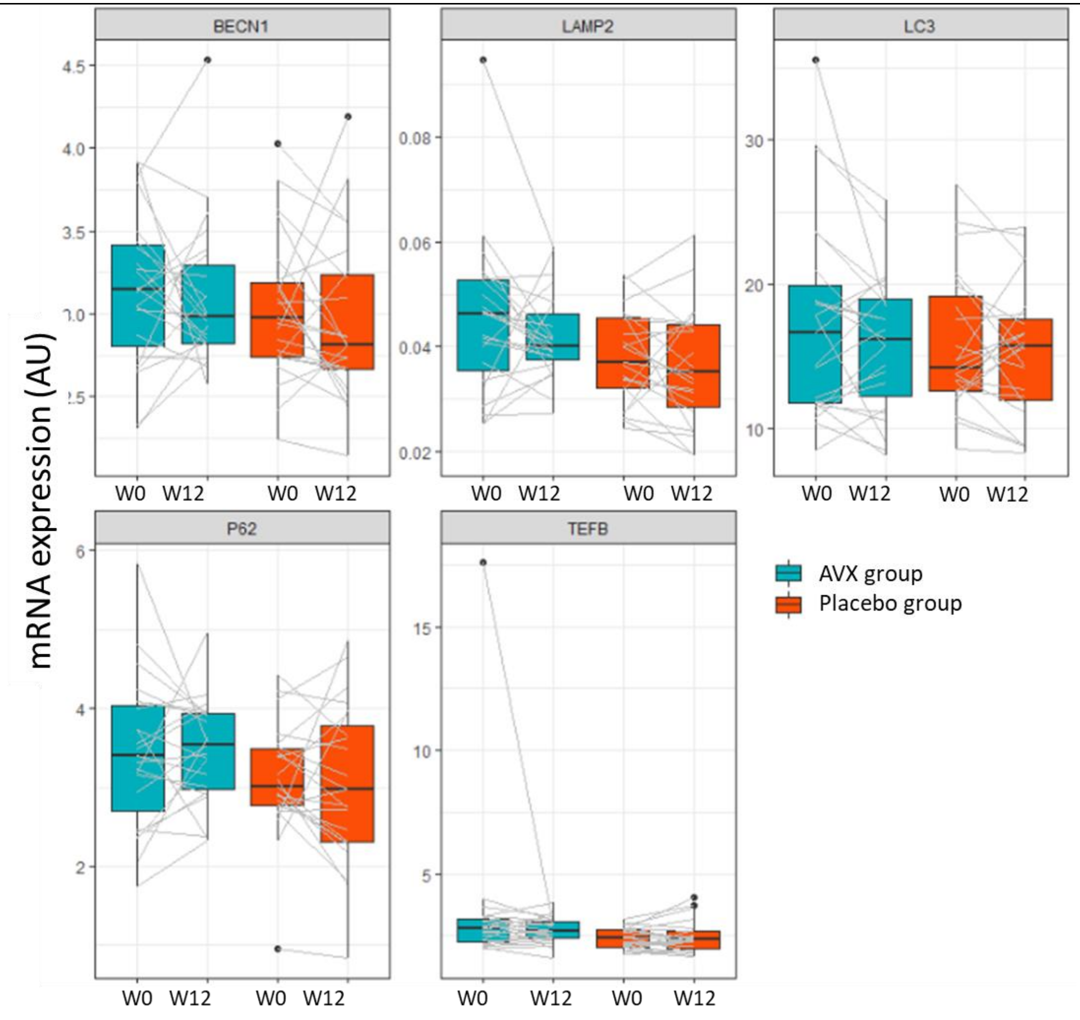

Figure S1 Autophagy genes expression before and after the 12-wk avocado extract (AvX) or placebo intervention in adults with obesity. Data are means ± SEMs; n = 23 and 23 for AvX and Placebo groups, respectively. Between-group analysis for the change from Week 0 to Week 12 for each variable was performed using one-way ANCOVA, adjusted for baseline value and sex.
